# Buccal oncocytoma: Report of a case and literature review

**DOI:** 10.1016/j.amsu.2019.05.016

**Published:** 2019-06-05

**Authors:** Sudha Shahi, Tika Ram Bhandari, Prakash Bahadur Thapa, Deependra Shrestha

**Affiliations:** a Department of Otorhinolaryngology Head and Neck Surgery, National Academy of Medical Sciences, Bir Hospital, Kathmandu, Nepal; b Department of General Surgery, People's Dental College and Hospital, Kathmandu, Nepal

**Keywords:** Buccal oncocytoma, Salivary gland

## Abstract

**Introduction:**

More common in major salivary glands, oncocytomas are very rare tumors. They commonly occur in the parotid gland and are painless slow growing predominantly benign tumors. The term “oncocytoma” was introduced by Jaffe to designate those tumors of the salivary glands that consist predominantly of oncocytic cells lining the salivary ducts (1) Similalry, Meza- Chavez had proposed the name “oxyphilic granular cell adenoma. (2) Oncocytomas are extremely rare, benign and slow growing in nature. Here we present a rare case of buccal oncocytoma which is to our knowledge the 19th case of intraoral minor salivary gland tumor and the 7th reported case of buccal oncocytoma.

**Case presentation:**

Here we present an exceedingly rare case of buccal oncocytoma in a 14 years boy who presented to the department of ENT with right buccal swelling for 6 months. He was posed the diagnosis of buccal cyst after cytological examination supported by CT scan. He then underwent an excisional biopsy where the final diagnosis was made as Buccal Oncocytoma.

**Conclusions:**

Though very rare in the picture, conditions like salivary gland oncocytomas still are reported on and off in the literature. The treatment of which is complete surgical excision.

## Highlights

• Buccal oncocytoma is one of the rare entities salivary gland oncocytoma.
• So far only 6 cases of buccal oncocytoma has been mentioned in literature.
• The definitive treatment is surgical excision.

## 1 Introduction

Oncocytoma is a benign tumor that consists predominantly of oncocytic cells lining the salivary ducts. The term “oncocytoma” was introduced by Jaffe [1] Similarly, Meza- Chavez had proposed the name “oxyphilic granular cell adenoma “due to a diffuse granular appearance of the cytoplasm of the tumor cells which is due to eosinophilic granular cytoplasm, due to increased numbers of mitochondria [2]. It is usually solitary, unilateral and occur commonly in women in their 7th or 8th decades of life [5]. Oncocytomas were originally identified in the salivary gland in 1931 by Hamperl. It had also been recognized in the thyroid and parathyroid glands. They were recognized in the kidneys in 1976 by Klein and Valensi [3,4]. Similarly, they account for <2% of all salivary gland neoplasms. They occur primarily in parotid glands with only a small percentage occurring in minor salivary glands [6]. Though they are bening and slow growing in nature, less than 1% have the chance of malignant transformation [7]. Fine needle aspiration cytology is usually not adequate for the diagnosis of buccal oncocytoma. Thus, CT scan and MRI are required in most of the cases. The final diagnosis is often made after a histopathological examination. Complete surgical excision of oncocytomas is the treatment of choice. Superficial or total parotidectomy may be required in cases of parotid gland oncocytomas. In cases of minor salivary gland oncocytomas, complete removal of the tumor is the treatment of choice.

The study above was done in compliance with the SCARE 2018 statement [8].

## 2 Case presentation

**Fig. 1:**
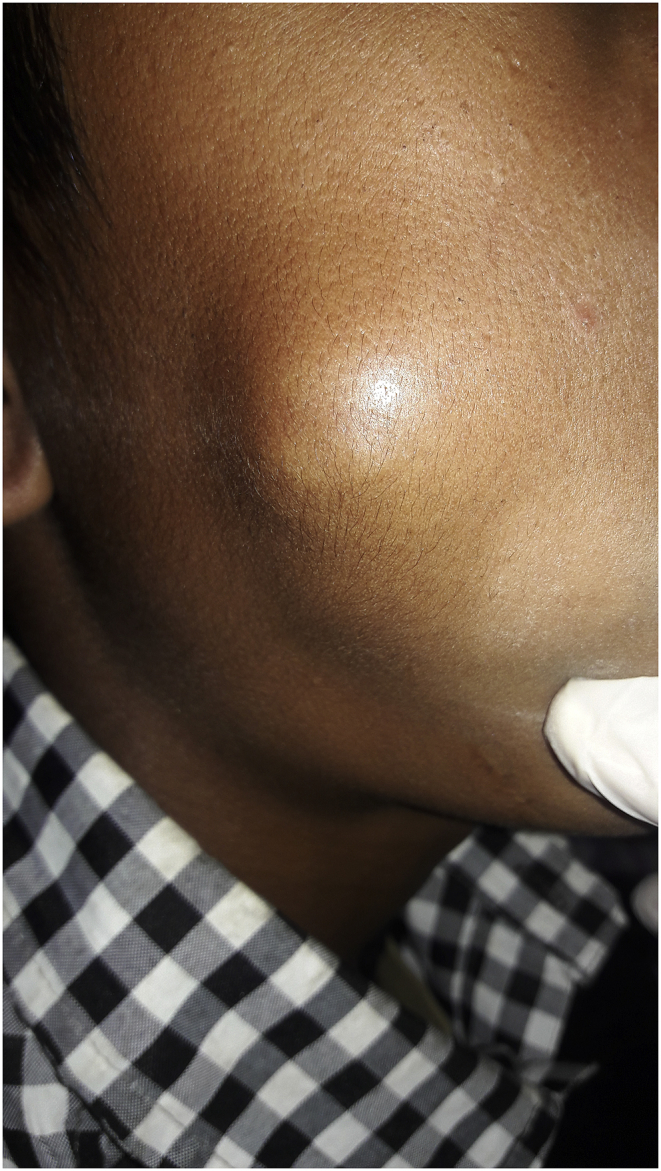
Swelling in the right buccal region.

**Fig. 2:**
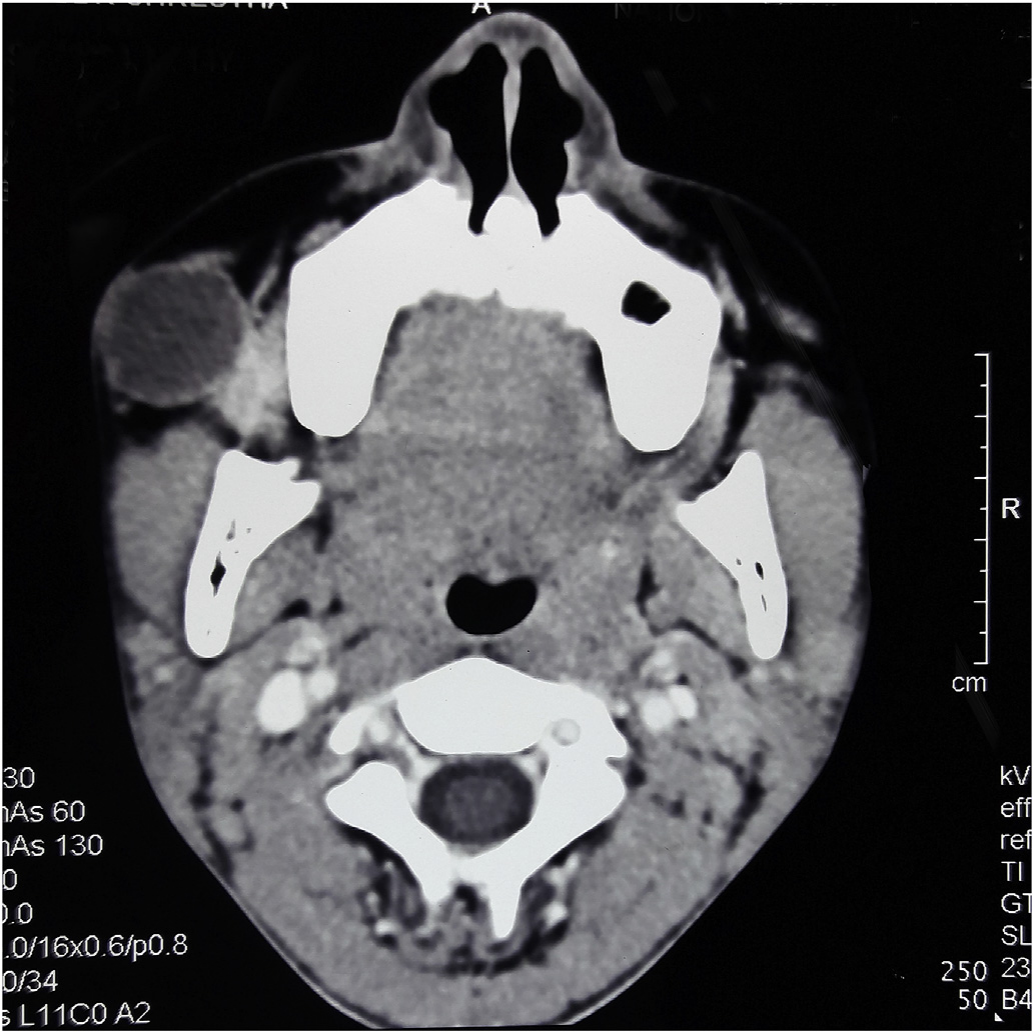
CT scan of head showing cystic lesion in the right buccal space.

**Fig. 3:**
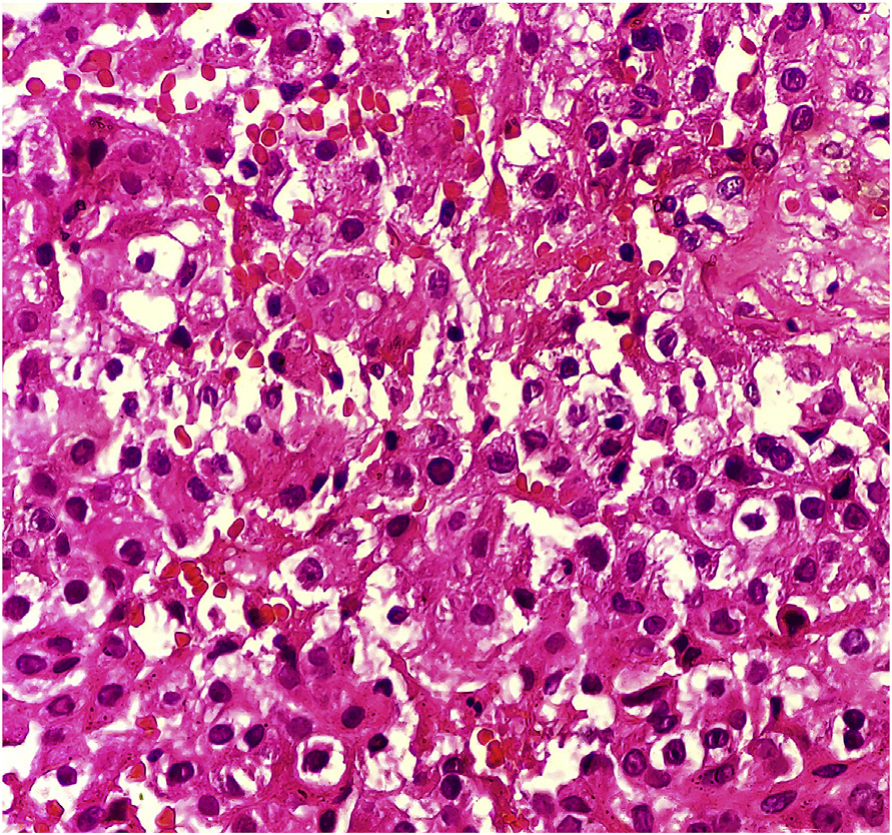
Histopathlogical slide of postoperative specimen.

Here we present an exceedingly rare case of buccal oncocytoma in a 14-year-old boy who presented to the Department of ORL Head and Neck Surgery with right buccal swelling for 6 months The swelling was progressively enlarging in size. There was no history of associated pain. There was no history of dysphagia. On examination, there was a single cystic swelling in right cheek around 2*2 cm^2^ with ill-defined margins (Fig. 1). It was nontender and palpable bimanually. Rest of the Oropharynx and laryngopharynx revealed no abnormality. There were no palpable neck nodes. His vitals were stable with normal blood parameters. He underwent a cytological examination of the mass which reported it as a chronic granulomatous lesion. CT scan of head and neck revealed well defined cystic lesion measuring 2.6*21*19 mm^3^ lesion in the right buccal space arising from the buccinator muscle and displacing the zygomaticus major (Fig. 2). With the findings above he was posed the provisional diagnosis of the right buccal cyst and planned for excision biopsy under General Anesthesia. Surgery was performed by our team of surgeons of ORL Head and Neck Surgeons. We preferred the intraoral approach. Intraoperatively there was a well defined cystic lesion measuring around 3*3 cm^2^ in the right buccal space. The cyst contained thick mucinous fluid. The specimen was sent for histopathological examination. Findings of the histopathological examination were consistent with oncocytoma (Fig. 3). He was kept on intravenous antibiotics. The postoperative period was uneventful with mild soft tissue swelling over the operated area without any collections. He was discharged on the 7th postoperative day. On subsequent follow up 1 week later, swelling over the buccal region had subsided. The intraoral surgical wound had healed. He was further followed up after 3 months. There were no signs of recurrence or disease progression.

## 3 Discussion

Salivary gland oncocytomas are extremely rare tumors. They are benign slow growing in nature. Oncocytomas in head and neck region are more common in major salivary glands as compared to a minor. They comprise of 0.5%–1.5% of all salivary gland tumors. Meanwhile, the incidence of salivary gland oncocytomas in the parotid gland accounts for 78%–84%. It is exceedingly rare in minor salivary glands which include sites like, lower lip, palate, pharynx, tonsillar fossa, buccal mucosa. So far only 18 cases of intraoral minor salivary gland oncocytomas have been reported in the literature. Out of the 18 cases, 6 cases are of buccal oncocytoma [6,9]. Thus here we report the 19th case of intraoral minor salivary gland tumor and the 7th of buccal oncocytoma. As discussed earlier, diagnosis is often challenging since Fine needle aspiration cytology doesn't provide enough information. CT scan, MRI can be helpful to delineate the nature and the location and plan for surgery but PET scans on the other hands have more roles in malignant oncocytomas with metastasis. Thus, a complete diagnosis is often made after a histopathological examination. Complete surgical excision of oncocytomas is the treatment of choice. In the case of parotid gland oncocytomas, superficial or total parotidectomy may be required. However, in case of other minor salivary gland oncocytomas, complete removal of the tumor is the treatment of choice. Likewise, in our case, we performed peroral excision of the tumor with good postoperative results. On subsequent follow up there was no sign of disease progression.

## 4 Conclusions

Though very rare in the picture, conditions like salivary gland oncocytomas still are reported on and off in the literature. More localized minor salivary gland tumors can be treated with complete excision while those related to parotid gland might require total or superficial parotidectomy. Thus we can say that the location of the tumor is determining factor in the treatment procedure.

## Ethical approval

As this is a case report, informed consent has been taken from the patient.

## Sources of funding

No funding was available for the article.

## Author contribution

1-Dr.Sudha Shahi –Concept design, Review, literature search, writing paper, final decision to publish.

2-Dr.Tika Ram Bhandari - Review, literature search, writing paper, final decision to publish.

3. Dr.Prakash Bahadur Thapa: Supervised the writing of the manuscript, final decision to publish.

4. Dr.Deependra Shrestha: Supervised the writing of the manuscript, final decision to publish.

## Conflicts of interest

The authors state no conflict of interests.

## Research registration number

None.

## Guarantor

Dr. Sudha Shahi.

## Consent for publication

Written informed consent was obtained from the patient for publication of this case report and any accompanying images. A copy of the written consent is available for review by the Editor-in-Chief of this journal.

## Registration of research studies

Is a case report.

## Provenance and peer review

Not commissioned externally peer reviewed.
